# Synthesis of Octacalcium Phosphate Containing Glutarate Ions with a High Incorporation Fraction

**DOI:** 10.3390/ma16010064

**Published:** 2022-12-21

**Authors:** Taishi Yokoi, Masahiro Watanabe, Tomoyo Goto, Sikun Meng, Tohru Sekino, Masaya Shimabukuro, Masakazu Kawashita

**Affiliations:** 1 Institute of Biomaterials and Bioengineering, Tokyo Medical and Dental University (TMDU), 2-3-10 Kanda-Surugadai, Chiyoda-ku, Tokyo 101-0062, Japan; 2 SANKEN (The Institute of Scientific and Industrial Research), Osaka University, 8-1 Mohogaoka, Ibaraki, Osaka 567-0047, Japan; 3 Institute for Advanced Co-Creation Studies, Osaka University, 1-1 Yamadaoka, Suita, Osaka 565-0871, Japan

**Keywords:** octacalcium phosphate, glutarate ion, incorporation

## Abstract

Octacalcium phosphate (OCP) has received considerable attention in the field of ceramic biomaterials as an advanced functional material. It exhibits a layered structure composed of apatitic and hydrated layers and can incorporate various dicarboxylate ions into the hydrated layer. Saturated dicarboxylic acids (HOOC(CH_2_)*_n_*COOH) with an odd number of methylene groups (–CH_2_–) exhibit lower incorporation fractions than those with an even number of methylene groups, possibly owing to a compositional dependence on the synthetic method. In this study, calcium carbonate, phosphoric acid, and various amounts of glutaric acid were used to produce glutarate-ion-incorporated OCP by a wet chemical method, which is different from the conventional synthetic strategy. While utilising 1–20 mmol of glutaric acid during synthesis did not produce the desired product, using 25 mmol of glutaric acid resulted in the formation of single-phase glutarate-ion-incorporated OCP with a Ca/P molar ratio of 1.57 and a 90% incorporation fraction of glutarate ions. This glutarate-ion-incorporation fraction is significantly higher than that reported in the previous studies (35%). Thus, the synthetic procedure proposed herein was able to produce single-phase OCP containing glutarate ions with a high incorporation fraction. Our findings can contribute to development of novel functional ceramic biomaterials in the future.

## 1. Introduction

Octacalcium phosphate (OCP, Ca_8_(HPO_4_)_2_(PO_4_)_4_·5H_2_O) has attracted immense attention in biomaterials research as a novel hard-tissue repair material [1]. Numerous studies report in vitro [2,3,4,5,6,7] and in vivo [8,9,10,11,12,13,14,15,16,17,18] biological characterisations and clinical tests of OCP-based biomaterials [19,20,21,22,23]. Composite materials composed of OCP and collagen exhibited excellent biological properties, which make them a good substitute for autogenous bone. Additionally, a theranostic material using organically modified OCP at the molecular level has been proposed [24]. Therefore, OCP is a promising material for the fabrication of next-generation advanced ceramic biomaterials.

OCP comprises a layered structure composed of apatitic and hydrated layers [25,26,27]; the hydrogen phosphate ions (HPO_4_^2−^) in the hydrated layer can be substituted by various carboxylate ions [28] (di-, tri-, and tetracarboxylate ions, but mainly dicarboxylate ions). OCP is the only calcium phosphate compound that enables the incorporation of carboxylate ions in its crystals. So far, no publications have reported on the synthesis of carboxylate-ion-incorporated OCP by soaking carboxylic-acid-free OCPs in aqueous carboxylic acid solutions, indicating that carboxylic acid incorporation into OCP crystals likely occurs during crystal growth—that is, during the OCP synthesis. Thus, numerous studies have attempted OCP synthesis with carboxylic acids to investigate this chemical specificity.

A typical synthetic method for carboxylate-ion-incorporated OCP involves the hydrolysis of alpha-tricalcium phosphate (TCP, Ca_3_(PO_4_)_2_) in an aqueous solution of the relevant carboxylic acid [29]. This method has been extensively used to investigate the incorporation of dicarboxylate ions into OCP [30,31,32,33,34]. The incorporation of dicarboxylic acid into OCP is challenging depending on the molecule to be incorporated; for instance, isophthalic acid has been successfully incorporated into OCP, but incorporation of terephthalic acid has not yet been achieved [33]. In addition, certain carboxylic acids, such as azelaic acid, have been reported to be incorporable by some researchers, while others report they cannot be incorporated [32,33]; this can be attributed to the differences in the synthetic conditions. Therefore, the investigation of synthetic methods is a vital aspect of research on carboxylate-ion-incorporated OCPs.

**Figure 1:**
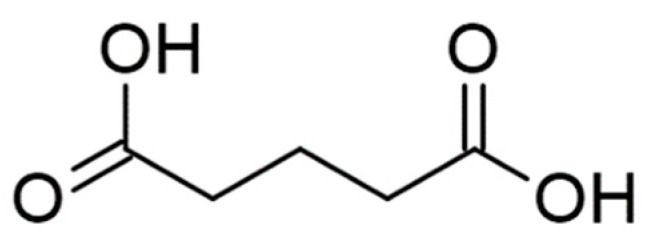
Molecular structure of glutaric acid.

Moreover, saturated dicarboxylic acids (HOOC(CH_2_)*_n_*COOH) with an odd number of main-chain methylene groups (–CH_2_–) exhibit a lower incorporation fraction in OCP than those with an even number of methylene groups [34]; this was first pointed out by Marković et al. in 1993 [32], this phenomenon requires further investigation. The low incorporation fraction of carboxylic acids with an odd number of methylene groups could also be attributed to a synthetic method dependence. We have studied and established the synthetic method of carboxylate-ion-incorporated OCP by the reaction of calcium carbonate and phosphoric acid in the carboxylic acid solution [35,36,37,38,39,40]. By synthesizing samples using this method, we have successfully obtained OCPs with precisely controlled interplanar spacings. In addition, while incorporating carboxylic acid into OCP, we found an elaborate molecular recognition between the OCP crystals and carboxylic acid molecules. However, so far, we have not attempted to incorporate the carboxylic acids with an odd number of methylene groups into OCP using this synthetic method. In this study, glutarate-ion-incorporated OCP was synthesised using our synthetic method instead of the conventional synthetic method that uses alpha-TCP. Glutaric acid is a typical carboxylic acid with an odd number of methylene groups (*n* = 3, Figure 1).

## 2. Materials and Methods

### 2.1. Synthesis

Carboxylate-ion-free OCP (plain OCP) and glutarate-ion-incorporated OCP were produced through a previously reported wet process [35]. To fabricate plain OCP, 6.0 mmol of H_3_PO_4_ (85% aqueous solution; Wako Pure Chemical Industries, Osaka, Japan) was added to 100 cm^3^ of ultrapure water at 60 °C under stirring (500 rpm), followed by the addition of 8.0 mmol of CaCO_3_ (calcite phase: 99.5%, Nacalai Tesque, Kyoto, Japan). After 3 h, the pH of the slurry was decreased to 5.0 by adding an appropriate amount of HCl solution (1.0 mol·dm^–3^, Nacalai Tesque). The aim of this process is to dissolve and remove the unreacted CaCO_3_. Subsequently, after 30 min of stirring at 60 °C, the precipitates were isolated by suction filtration using a conventional filter paper, rinsed with ultrapure water and ethanol, and dried at 40 °C overnight. This sample was labelled as the control.

Similarly, glutarate-ion-incorporated OCP was fabricated using a previously published method with appropriate modifications [35]. First, glutaric acid (1–25 mmol) (HOOC(CH_2_)_3_COOH: 99.0%, Tokyo Chemical Industry Co., Ltd., Tokyo, Japan) was completely dissolved in 100 cm^3^ of ultrapure water at 60 °C by adding NH_3_ solution (28 mass% aqueous solution, Wako Pure Chemical Industries), and the pH of the solution was adjusted to 5.5. Subsequently, an H_3_PO_4_ solution (5.0 mmol) was added to the glutaric acid solution, followed by the addition of 8.0 mmol of CaCO_3_. The slurry was stirred at 500 rpm at 60 °C. After 3 h, the pH of the slurry was reduced to 5.0 using an HCl solution, and the precipitates were isolated by suction filtration, rinsed, and dried after 30 min of stirring. These samples were labelled as GA*X*, where *X* is the amount (mmol) of glutaric acid used for synthesis.

### 2.2. Characterisation

The crystalline phases of the samples were characterised by powder X-ray diffraction (XRD; MiniFlex600, Rigaku Corp., Tokyo, Japan) using Cu Kα radiation and an X-ray wavelength of 0.154056 nm. The interplanar spacing of the (100) (*d*_100_) planes of the representative OCP samples was evaluated by XRD after mixing fluorophlogopite (Topy Industries Ltd., Tokyo, Japan) as an angular standard at an OCP: fluorophlogopite mass ratio of 4:1. The chemical structures of the typical samples were characterised by Fourier transform infrared (FTIR) spectroscopy (FT/IR-6200, JASCO Corp., Tokyo, Japan) using the KBr pellet method with an OCP:KBr (for infrared spectrophotometry, Wako Pure Chemical Industries) mass ratio of 1:300. The Ca/P molar ratios of the representative samples were determined using the inductively coupled plasma atomic emission spectroscopy (ICP-AES; ICP-8100, Shimadzu Co., Kyoto, Japan) after dissolving the samples in aqua regia. The carbon contents of the samples were evaluated using a carbon and sulfur analyser (EMIA-920V, HORIBA, Ltd., Kyoto, Japan), and their crystal morphologies were analysed using scanning electron microscopy (SEM, JSM-7900F, JEOL Ltd., Tokyo, Japan) after coating with a thin Pt film.

## 3. Results and Discussion

**Figure 2:**
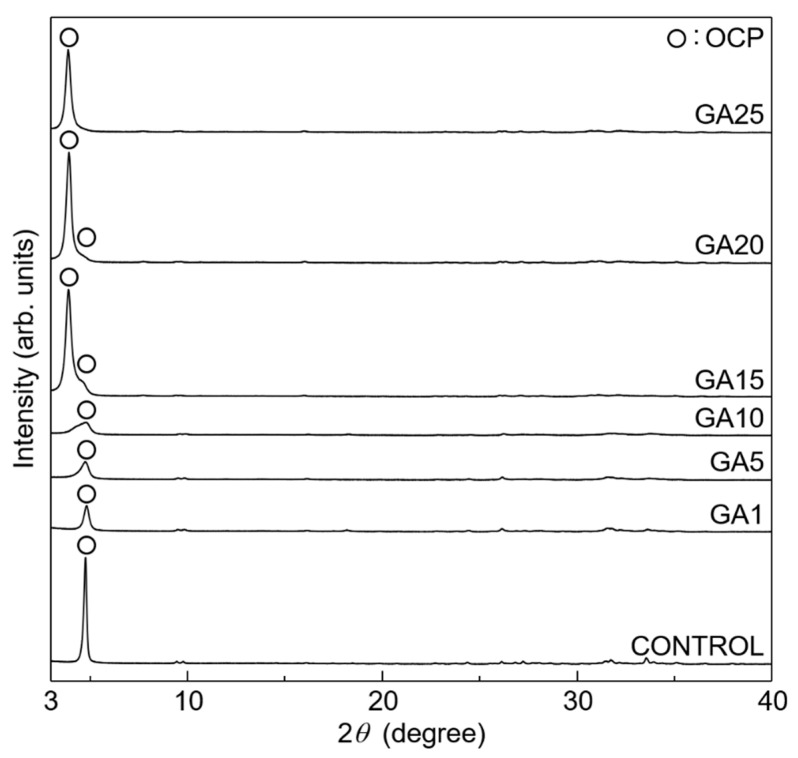
XRD patterns of the OCP samples synthesised using 1–25 mmol of glutaric acid as compared to plain OCP (control).

The XRD patterns of the specimens synthesised using different amounts of glutaric acid are shown in Figure 2. All the samples exhibited the same crystalline phase as OCP; other crystalline phases, such as those of the starting material calcite, were not detected. The control exhibited the crystalline phase of plain OCP, which was identified using the powder diffraction file (PDF) #01-074-1301. GA1 and GA5 also exhibited crystalline phases similar to that of plain OCP; however, the reflection peak intensities were lower than those of the control, indicating a disordered OCP-phase layered structure. 

The XRD peak positions of GA10 were almost identical to those of plain OCP, with an additional shoulder at the low-angle side of the main peak (at 4.7°); contrarily, GA15 exhibited a remarkable peak shift with a higher main peak intensity (at 3.9°). Thus, glutarate ions were incorporated into OCP and formed OCP with a well-ordered layered structure. The *d*_100_ calculated from the 2*θ* value of the main peak position was almost equal to that evaluated from the glutarate-ion-incorporated OCP, as described later in the paper. A small shoulder derived from plain OCP appeared at the higher-angle side of the main peaks in the GA15 and GA20 patterns, indicating that GA15 and GA20 were mixtures of plain and glutarate-ion-incorporated OCP. Moreover, this shoulder peak was absent in the GA25 pattern. Thus, 25 mmol of glutaric acid was required for the single-phase formation of glutarate-ion-incorporated OCP. Subsequently, the control and GA25 were characterised.

**Figure 3:**
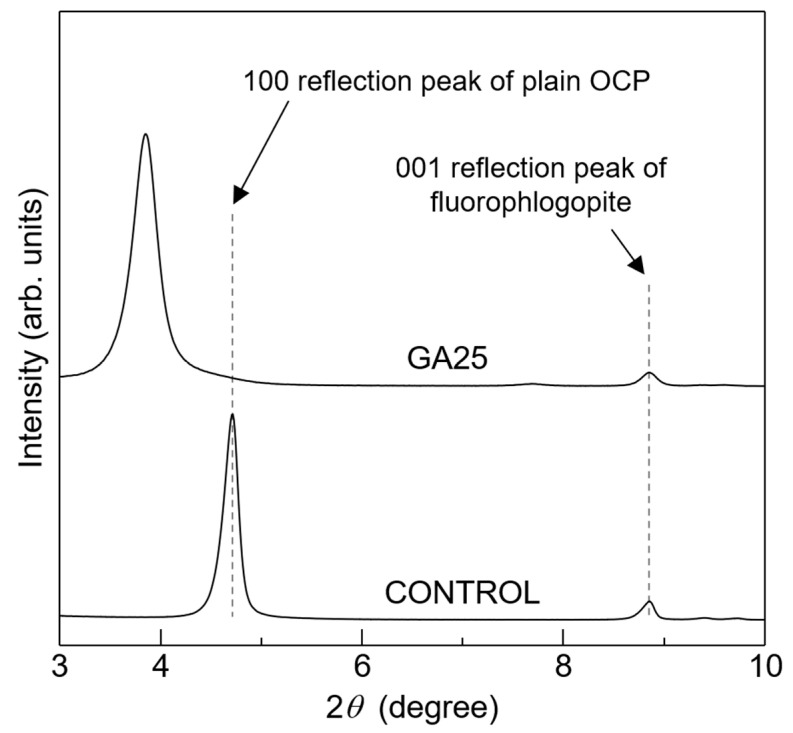
XRD patterns of plain OCP (control) and the OCP sample synthesised using 25 mmol of glutaric acid (GA25) with fluorophlogopite (an angular standard material).

The XRD patterns of the control and GA25 with the internal angular standard material fluorophlogopite (Figure 3) were used to determine the exact *d*_100_ value. The 100 reflection peak of the control was detected at 4.71°, and its *d*_100_ was calculated to be 1.87 nm; this value is almost equal to the *d*_100_ of plain OCP (1.878 nm). The 100 reflection peak of GA25 was detected at 3.85°, and its *d*_100_ was calculated to be 2.29 nm; this value is slightly higher than the experimentally determined and computationally predicted *d*_100_ values of glutarate-ion-incorporated OCP (2.23 nm [34] and 2.24 nm [41], respectively). This is possibly due to the higher incorporation fraction of glutaric acid in GA25 in this study compared to previous publications, as described below.

**Figure 4:**
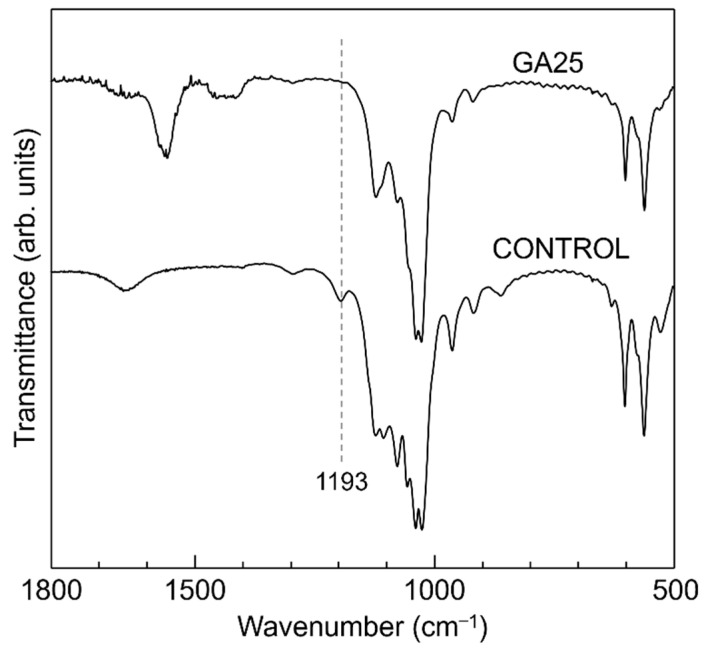
FTIR spectra of plain OCP (control) and the OCP sample synthesised using 25 mmol of glutaric acid (GA25).

The incorporation of glutarate ions into OCP was also confirmed by FTIR spectroscopy. The FTIR spectra of the control and GA25 are shown in Figure 4; the absorption peaks were assigned based on a previous report [42]. All the peaks in the control spectrum could be attributed to plain OCP. The peak derived from the hydrated-layer hydrogen phosphate ions was clearly detected at 1193 cm^–1^ in the control (indicated by a dashed line in Figure 4); however, this peak was absent in the GA25 spectrum, which exhibited absorption peaks derived from dissociated carboxyl groups in the range of 1600–1400 cm^–1^. This indicated a replacement of the hydrated-layer hydrogen phosphate ions in OCP by the glutarate ions in GA25. Therefore, the XRD and FTIR results confirmed GA25 to be a glutarate-ion-incorporated OCP. 

**Figure 5:**
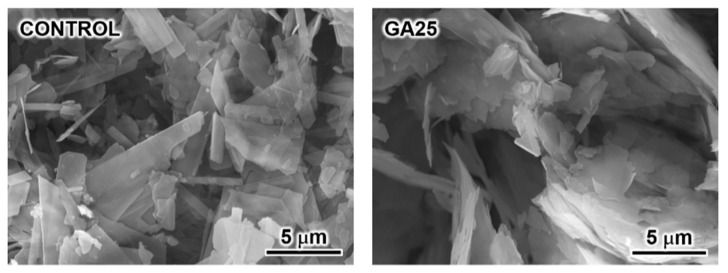
SEM images of plain OCP (control) and the OCP sample synthesised using 25 mmol of glutaric acid (GA25).

Figure 5 shows SEM images of the control and GA25. The control exhibited a plate-like shape that was several micrometres in size; this is the representative morphology of plain OCP. GA25 also exhibited a plate-like shape, with a slightly larger size than that of the control. This is consistent with previous reports on succinate- and suberate-ion-incorporated OCP [40]. Considering the crystal morphology in indicating the growth behaviour, the process of glutarate ion incorporation into OCP is possibly similar to the previously reported processes of succinate and suberate ion incorporation into OCP.

The compositional analysis indicated that the Ca/P ratios of the control and GA25 were 1.37 and 1.57, respectively, and their carbon contents were 0.1 and 4.7 in mass%, respectively. The general chemical formula of the OCP with incorporated dicarboxylate ions is Ca_8_(HPO_4_)_2–*z*_(DCI)*_z_*(PO_4_)_4_·*m*H_2_O (0 ≤ *Z* ≤ 1), where the DCI is dicarboxylate ion. As the Ca/P molar ratio of GA25 was 1.57, *z* in the chemical formula of OCP with incorporated dicarboxylate ions was calculated to be 0.90. Hence, the substitution fraction of hydrogen phosphate ions by glutarate ion in GA25, evaluated using its Ca/P ratio, was 90%.

Table 1 summarises the Ca/P molar ratio of the control and GA25 samples and the substitution fraction of GA25, along with the data previously reported by Monma [33]. According to a previous report by Monma [33], carboxylate ions of saturated dicarboxylic acids with an odd number of methylene groups in the main chain, such as malonate, glutarate, pimerate, and azelate ions, exhibit a lower incorporation fraction into OCP than those with an even number of methylene groups. Interestingly, the synthetic strategy proposed here fabricated OCP with a substitution fraction of 90%. In this study, the glutaric acid concentration in GA25 was approximately 250 mmol·dm^–3^, similar to the dicarboxylic acid concentration used in Monma’s process; thus, the high substitution fraction of glutarate ions could not be attributed to a higher reaction-solution glutaric acid concentration. However, the starting materials of the two synthetic methods were different. Here, the starting materials, calcium carbonate and phosphoric acid (used as the calcium and phosphate source, respectively), reacted to form dicalcium phosphate dihydrate (DCPD, CaHPO_4_·2H_2_O), which subsequently transformed into the OCP phase. In Monma’s process, alpha-TCP was used as the starting material, which transformed into the OCP phase.

Further research is required to understand the influence of the OCP-phase formation process on the final product composition. However, this study confirms that the substitution fraction of dicarboxylate ions with an odd number of methylene groups into OCP can be effectively increased by controlling the OCP-formation route. Glutamic acid, which is a derivative of glutaric acid, is a type of amino acid comprising an odd number of methylene groups. Glutamic acid enhances the osteoblast activation and extra-cellular matrix mineralization processes [43]; therefore, this synthetic process can effectively in incorporate such biologically relevant molecules into OCP. In addition, calcium phosphate nanoparticles have been investigated for applying drug carriers [44,45]. Thus, this study contributes immensely to the synthetic chemistry of dicarboxylate-ion-incorporated OCP and could facilitate the development of novel functional ceramic biomaterials.

## 4. Conclusions

In this study, glutarate-ion-incorporated OCP was synthesised via the reaction of CaCO_3_ and H_3_PO_4_ with different concentrations of glutaric acid. Plain OCP was formed with 1–10 mmol of glutaric acid, a mixture of plain and glutaric-acid-incorporated OCP was formed with 15–20 mmol of glutaric acid, and a single phase of glutarate-ion-incorporated OCP was formed with 25 mmol of glutaric acid. This glutarate-ion-incorporated OCP exhibited a plate-like shape that was several micrometres in size. FTIR analysis confirmed glutarate ion incorporation, with its fraction quantitatively evaluated by compositional analysis. The glutarate-ion-incorporated OCP exhibited a Ca/P molar ratio of 1.57, indicating the substitution fraction of replaceable hydrogen phosphate ions in OCP by glutarate ions to be 90%; this substitution fraction is higher than previously reported values. Hence, the synthetic strategy reported here effectively produces glutarate-ion-incorporated OCP with a high incorporation fraction. Moreover, our findings can contribute to the development of novel functional ceramic biomaterials.

## Figures and Tables

**Table 1:** Ca/P molar ratios and substitution fractions of the hydrated-layer hydrogen phosphate ions by dicarboxylate ions in the plain OCP (control) and the OCP sample synthesised using 25 mmol of glutaric acid (GA25), along with the previously reported data [33].

Incorporated Anion	Ca/P Molar Ratio	Substitution Fraction ^1^ (%)
Hydrogen phosphate ion (control)	1.37	N/A
Glutarate ion (GA25)	1.57	90 ^2^
Malonate ion	1.47 ^3^	42 ^3^
Succinate ion	1.55 ^3^	83 ^3^
Glutarate ion	1.45 ^3^	35 ^3^
Adipate ion	1.56 ^3^	86 ^3^
Pimerate ion	1.41 ^3^	22 ^3^
Suberate ion	1.55 ^3^	92 ^3^
Azelate ion	1.45 ^3^	50 ^3^
Sebacate ion	1.53 ^3^	79 ^3^

^1^ Substitution fraction indicates the replacement ratio of the replaceable HPO_4_^2–^ in OCP by dicarboxylate ions. ^2^ The substitution fraction of GA25 is calculated from its Ca/P molar ratio. ^3^ The values are cited from ref. [33].

## Data Availability

The data will be available on request.
